# The Parkinson’s disease-linked Leucine-rich repeat kinase 2 (LRRK2) is required for insulin-stimulated translocation of GLUT4

**DOI:** 10.1038/s41598-019-40808-y

**Published:** 2019-03-14

**Authors:** Natalja Funk, Marita Munz, Thomas Ott, Kathrin Brockmann, Andrea Wenninger-Weinzierl, Ralf Kühn, Daniela Vogt-Weisenhorn, Florian Giesert, Wolfgang Wurst, Thomas Gasser, Saskia Biskup

**Affiliations:** 1 Hertie Institute for Clinical Brain Research and German Center for Neurodegenerative Diseases, University Clinic Tuebingen, Tuebingen, Germany; 2 IZKF Facility for Transgenic Animals, Institute of Medical Genetics and Applied Genomics, University of Tuebingen, Tuebingen, Germany; 3 Max-Delbrueck-Center for Moleculare Medizin and Berlin Institute of Health, Berlin, Germany; 4 Helmholtz Zentrum Muenchen, Technical University Muenchen-Weihenstephan, Institute of Developmental Genetics, Neuherberg, Germany; 5 German Center for Neurodegenerative Diseases, Munich, Munich Cluster for Systems Neurology (SyNergy), Munich, Germany

## Abstract

Mutations within Leucine-rich repeat kinase 2 (LRRK2) are associated with late-onset Parkinson’s disease. The physiological function of LRRK2 and molecular mechanism underlying the pathogenic role of LRRK2 mutations remain uncertain. Here, we investigated the role of LRRK2 in intracellular signal transduction. We find that deficiency of Lrrk2 in rodents affects insulin-dependent translocation of glucose transporter type 4 (GLUT4). This deficit is restored during aging by prolonged insulin-dependent activation of protein kinase B (PKB, Akt) and Akt substrate of 160 kDa (AS160), and is compensated by elevated basal expression of GLUT4 on the cell surface. Furthermore, we find a crucial role of Rab10 phosphorylation by LRRK2 for efficient insulin signal transduction. Translating our findings into human cell lines, we find comparable molecular alterations in fibroblasts from Parkinson’s patients with the known pathogenic G2019S LRRK2 mutation. Our results highlight the role of LRRK2 in insulin-dependent signalling with potential therapeutic implications.

## Introduction

Leucine-rich repeat kinase 2 (LRRK2) in humans is encoded by the *PARK8/LRRK2* gene^1,2^. Genetic variations within the *LRRK2* gene are linked to a number of diseases, including Parkinson’s disease (PD), Crohn´s disease and Hansen’s disease^3–8^. PD is the most common movement disorder, affecting ≈1% of the population over the age of 60, increasing to 4% at 80 years of age^9^. The neuropathological hallmarks of PD are progressive neurodegeneration of dopaminergic (DA) neurons in the substantia nigra (SN) and the wide spreading of neuronal intracellular inclusions known as Lewy bodies with the cardinal component being alpha-synuclein^10^. Dominantly inherited mutations in *LRRK2* are the most common cause of familiar PD and account for up to 2% of sporadic late-onset Parkinsonism^11,12^.

The *LRRK2* gene encodes a large multi-domain protein kinase containing an ankyrin-like- and a leucine-rich- repeat region, a Rab-like GTPase domain, a tyrosine-kinase-like domain and a WD-like domain. LRRK2 appears to regulate a variety of cellular processes critical for homeostasis and cell survival. LRRK2 has been shown to control neurite morphology and complexity^13–19^, to regulate synaptic vesicle recycling/endocytosis^20–25^ and dopamine receptor trafficking^26^. Furthermore, LRRK2 has been linked to the intertwined pathways regulating inflammation, protein degradation, mitochondrial- and autophagy/lysosomal functions^17,27–34^. It interacts with and affects microtubule and actin structure and dynamics^35,36^. A number of proteins including but not restricted to Ras related proteins Rab3, Rab5, Rab7, Rab8, Rab10, Rab12 and Rab32 (summarized in)^17,37–40^ as well as p21-activated kinase 6^13^, EndophilinA^25^, Rac1^15^, NFAT^33^, ezrin/radixin/moesin (ERM) family proteins^36^, Sec16A^41^, Akt1^42^, 14-3-3 proteins^43–49^, Snapin^50^ and others have been described as putative substrates and interactors of LRRK2 *in vivo* and/or *in vitro*. However, the molecular mechanism behind physiological function of LRRK2, also with respect to its mutated forms, remains poorly understood.

Here, we investigated the physiological role of LRRK2 in intracellular signalling using Lrrk2 deficient rat and mice models. We analysed neurite outgrowth and survival of postnatal primary hippocampal neurons in the presence of different growth factors and intracellular signal transduction in monocytes and fibroblasts as response to diverse extracellular ligands. We find that deficiency of Lrrk2 affects selectively insulin-dependent intracellular signalling. After stimulation with insulin the rapid intracellular translocation of glucose transporter type 4 (GLUT4) to the cell surface fails in Lrrk2 deficient cells from 6 months old animals. This malfunction is accompanied by slight elevation of protein kinase B (PKB, Akt) phosphorylation. Furthermore, we find that this defect is restored during aging by prolonged insulin-dependent activation of Akt and Akt substrate of 160 kDa (AS160/TBC1D4), and is compensated by elevated basal levels of GLUT4 on the plasma membrane. Interestingly, we find comparable molecular dys-regulations in fibroblasts from Parkinson’s patients with G2019S mutated LRRK2. In addition, we find LRRK2 dependent phosphorylation of Rab10 as well as dynamic insulin-triggered changes in Rab10 phosphorylation. Collectively, our data demonstrates that the Parkinson’s disease-linked LRRK2 plays a crucial role in insulin-driven translocation and/or fusion of GLUT4-vesicles to the plasma membrane through the phosphorylation of Rab10.

## Results

### Lrrk2 deficient animal models

**Figure 1:**
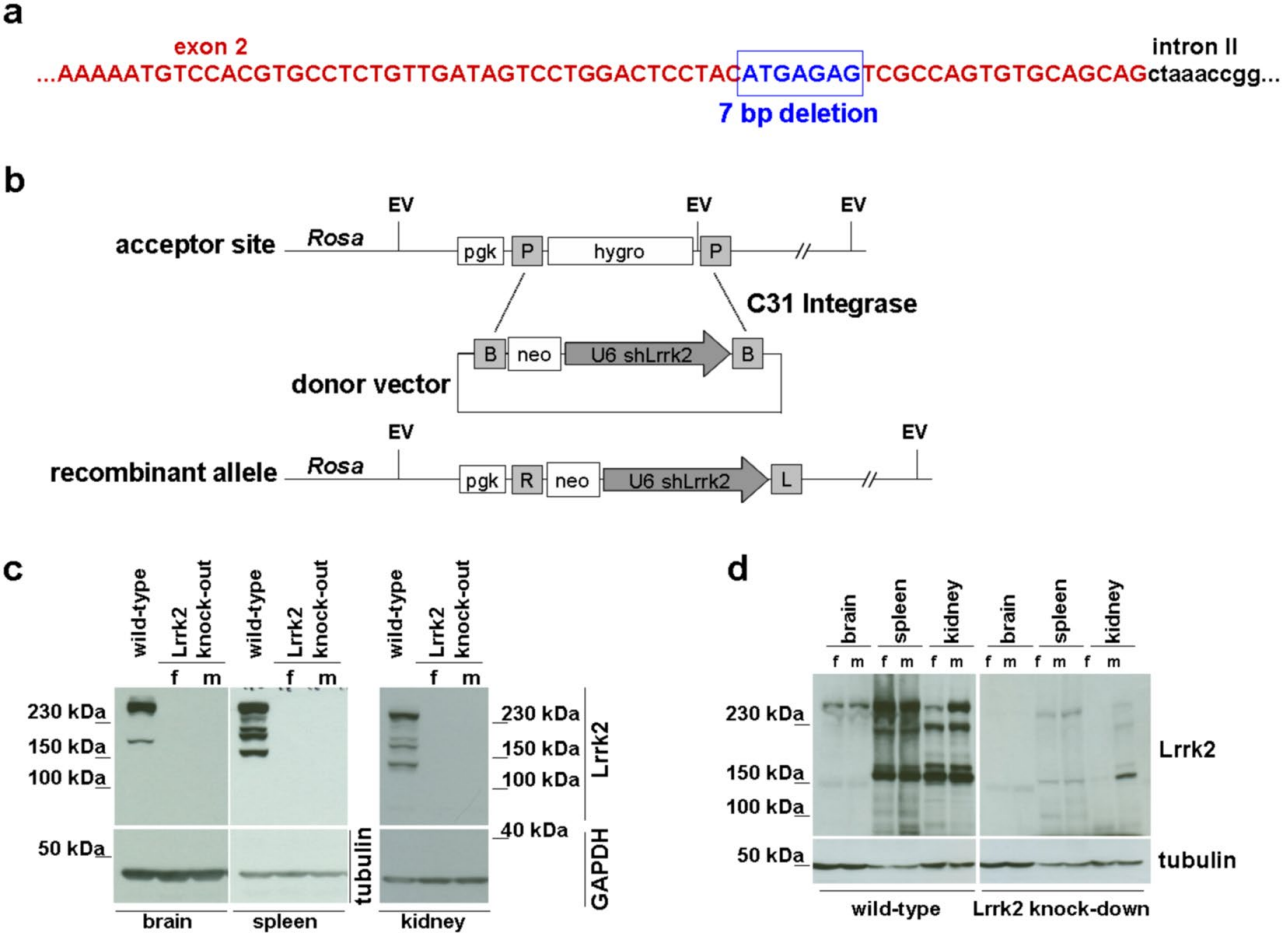
Animal models. (**a**) Schematic representation of 7 bp deletion (blue) in genome of Lrrk2 deficient rat line. A part of exon 2 (red) and intron II (black) are depicted for orientation. (**b**) Generation strategy for Lrrk2 knock-down mouse line carrying a shLrrk2 transgene inserted into the *Rosa* locus. (**c**) Western blot analysis of Lrrk2 expression in brain-, spleen- and kidney- protein extracts of Lrrk2 deficient rats and (**d**) Western blot analysis of Lrrk2 expression in different tissues (brain, spleen and kidney) of Lrrk2 knock-down mouse in comparison to wild-type using anti-LRRK2 MJFF#2 antibody. f: female; m: male.

To elucidate the physiological impact of LRRK2 deficiency, we used three different animal models lacking Lrrk2 protein expression: a Lrrk2 knock-out rat line with a 7 bp deletion in exon 2 generated by us (Fig. 1a), a Lrrk2 knock-out mouse line with a deletion of partial exon 39 and complete loss of exon 40 generated by the T. Dawson lab, Baltimore, USA^51^ and a Lrrk2 knock-down mouse line carrying a shLrrk2 transgene inserted into the *Rosa* locus (Fig. 1b) generated by the W. Wurst lab, Munich, Germany^52^. The protein expression analysis shows a complete absence of the full length Lrrk2 protein and its splicing forms in different tissues in the Lrrk2 knock-out rat (Fig. 1c) and mouse^51^, and a strong reduction (over 90%) of Lrrk2 protein expression in Lrrk2 knock-down mouse (Fig. 1d).

### LRRK2 influences cell survival and neurite outgrowth in a growth factor independent manner

**Figure 2:**
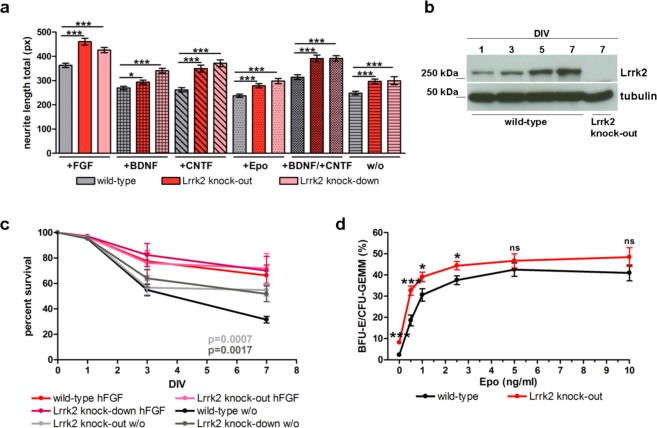
Effect of different growth factors on neurite outgrowth and cell survival. (**a**) Neurite length of primary hippocampal neurons from wild-type and Lrrk2 deficient mice lines Lrrk2 knock-out and Lrrk2 knock-down at DIV3 in presence of different growth factors (hFGF, BDNF, CNTF, Epo and without any growth factors (w/o); mean and SEM). (**b**) Lrrk2 expression in mouse wild-type primary hippocampal neurons at DIV 1 to 7 (lanes 1 to 4) and Lrrk2 deficient neurons at DIV 7 (lane 5). Lrrk2 expression was detected using the LRRK2 specific antibody MJFF#2. (**c**) Survival of Lrrk2 deficient hippocampal neurons (Lrrk2 knock-out and Lrrk2 knock-down) in comparison to wild-type neurons at DIV 1, 3 and 7 in presence of hFGF and without addition of any growth factors (w/o) as negative control (mean and SEM). (**d**) Percentage of growing late erythrocyte progenitor colonies (BFU-Es) and erythrocyte progenitor containing mix colonies (CFU-GEMMs) in CFC-assay media with different Epo concentrations (0, 0.5, 1, 2.5, 5 and 10 ng/ml; mean and SEM).

It has been shown previously that Lrrk2 deficiency impairs neurite outgrowth, morphology and neuronal survival^16,17,19,36^. Several growth factors are known as important regulators of these processes. To investigate the possible function of LRRK2 in context with intracellular signalling we analysed neurite outgrowth and survival of postnatal primary hippocampal neurons from Lrrk2 deficient mouse lines in the presence of different growth factors (human fibroblast growth factor (hFGF), brain-derived neurotrophic factor (BDNF), glial cell line-derived neurotrophic factor (CNTF), Erythropoetin (Epo) and without any growth factor as negative control by high throughput microscopy (Suppl. Fig. 1b,c). We found, that independent of which growth factor was added, or even without the addition of any growth factors, hippocampal neurons from both deficient mouse lines show a strong increase in neurite outgrowth in comparison to cells from wild-type littermates. These differences achieve a significant value on day *in vitro* DIV 1 (Suppl. Fig. 1a) and persist to day 3 (Fig. 2a). Lrrk2 protein expression in culture at DIV 1 and later time points was ensured by Western blotting (Fig. 2b). Furthermore, in comparison to wild-type control cells we found a significantly better survival of Lrrk2 deficient hippocampal neurons in media without any growth factors (Fig. 2c). We hypothesized that LRRK2 is involved as a kind of negative regulator in general higher ranked intracellular activity important for cell survival and other cellular processes such as neurite maintenance and speculated that this LRRK2 function is not restricted to neuronal tissue.

In addition to nervous system LRRK2 protein is expressed in several blood cell types^33,53^. The formation of blood cellular components (hematopoiesis) is a highly complex and well studied process: the development of different blood cell types undergoes a series of cytokine and growth factors dependent steps that we aimed to investigate with respect to Lrrk2 deficiency. Using a colony forming cell (CFC) assay we compared the development of different blood cell progenitors in 3–4 months old Lrrk2 deficient and wild-type mice. Most interestingly, we found a significantly higher Erythropoietin (Epo) independent survival of erythrocyte progenitors in Lrrk2 deficient mice (Fig. 2d). The most striking difference was seen in the presence of low and middle range Epo concentrations from up to 2.5 ng/ml medium, pointing towards an Epo independent additional survival mechanism activated by Lrrk2 deficiency.

### Lrrk2 deficiency impairs feedback control of insulin signalling in aged cells

**Figure 3:**
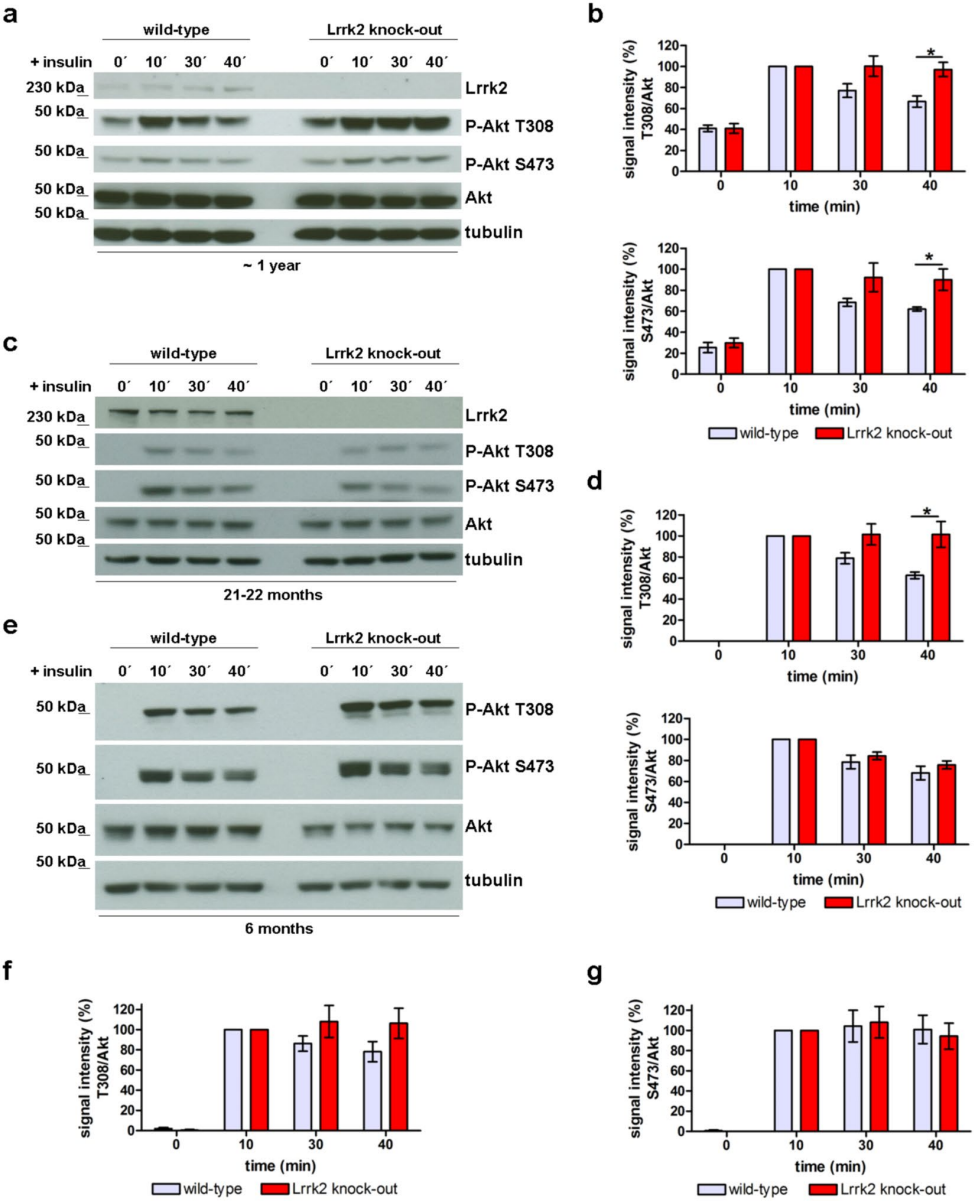
Induced phosphorylation of Akt in Lrrk2 deficient cells. (**a**) Western blot analysis of P-Akt Thr 308 and Ser473 in protein extracts from monocytes of 1 year old Lrrk2 deficient and wild-type mice stimulated with insulin at different time points (0 to 40 min) after stimulation. (**b**) Quantification of P-Akt Thr308 (b, top) and P-Akt Ser473 (b, button) intensity in protein extracts from monocytes of 1 year old Lrrk2 deficient and wild-type mice at different time-points after stimulation with insulin. N = 8 (for Thr308) and 6 (for Ser 473) independent experiments, normalized to Akt, mean ± SEM. (**c**) Western blot analysis of protein lysates from fibroblasts of 22 months old Lrrk2 deficient and wild-type rats at different time points after stimulation with insulin and (**d**) the corresponding quantification of P-Akt Thr308 (top) and P-Akt S473 (button) signal intensity. N = 10 independent experiments, normalized to Akt, mean ± SEM. (**e**) Western blot analysis of protein lysates from fibroblasts of 6 months old rats (wild-type and Lrrk2-deficient) after stimulation with insulin and corresponding quantification of P-Akt Thr308 (**f**) and S473 (**g**) signal intensity. N = 7 independent experiments, normalized to Akt, mean and SEM.

Serine/threonine protein kinase B (=Akt) serves as the most central downstream player of important signalling pathways. Defective Akt is implicated as a putative signalling pathway linked to loss of dopaminergic neurons in PD^54^. We asked about age-related dynamic phosphorylation of Akt in Lrrk2 deficient animals as response to different extracellular stimuli. Primary hippocampal neurons can be isolated and cultured from embryonic and early postnatal mice, but not from old mice (~1 year of age). Therefore, we went on to characterize blood derived cells with respect to this question. Monocytic cells are a suitable model for this study because they can be activated by a variety of extracellular factors and LRRK2 has been shown to be highly expressed in blood cells such as monocytes and others^33,53^. In order to study aging-related dynamic phosphorylation of Akt, monocytes from “young” (5 months) and “old” (~1 year) Lrrk2 deficient mice and wild-type littermates were isolated and stimulated with Interleukin 3 (IL-3), Interleukin 6 (IL-6), macrophage colony stimulating factor (M-CSF), insulin or complement component 5a (C5a). The phosphorylation on positions Thr308 and Ser473 of Akt at defined time points after stimulation was analysed by immunoblotting. In wild-type monocytes, the phosphorylation of Akt reach a maximum after activation of corresponding signalling pathways by binding of an extracellular ligand to the respective receptor and subsequently is decreased by dephosphorylation. Here, we found an aging-related continuous activation of Akt in monocytes of ~1 year old (but not 5 months old) Lrrk2 deficient mice in response to stimulation with the hormone insulin, as reflected by a permanent phosphorylation of Akt on positions Thr308 (p-value 0.013, Fig. 3a,b (top)) and Ser473 (p-value 0.045, Fig. 3a,b (button)) at time-point 40 min (s. also Suppl. Fig. 2a,b). This insulin-dependent alteration in Akt phosphorylation is not restricted to monocytic cells: we further confirmed our data in fibroblasts from ~1 year old Lrrk2 deficient mice (Suppl. Fig. 3c,d). Furthermore, to exclude an animal-model dependent unspecific observations, we analysed the insulin-dependent Akt phosphorylation in fibroblasts from “young” (6 months old) and “old” (~22 months old) Lrrk2 deficient rats generated in our lab (Fig. 1a,c). We found a significant (p-value 0.002 at 40 min) dys-regulation of Akt-Thr308 phosphorylation in cells from 22 months old Lrrk2 knock-out rats (Fig. 3c,d (top)) comparable with our data from Lrrk2 deficient mice (Fig. 3a,b). The dynamics of insulin-triggered phosphorylation of Akt in cells from 6 months old animals as well as the phosphorylation on Akt-Ser473 in cells from both age groups tested was not significant affected in Lrrk2 deficient rats (Fig. 3c,d (button), e–g).

The Lrrk2-dependent defective feedback regulation of Akt (especially Akt-Thr308) seems to be restricted to the hormone insulin: the dynamics of Thr308 Akt phosphorylation induced by hFGF, IL-3, IL-6, M-CSF and anaphylatoxin C5a is not visibly affected in Lrrk2 deficient cells as demonstrated by Western blot analysis (Suppl. Figs 2c–g and 3a,b). Therefore we investigated the physiological consequence of Lrrk2 deficiency for neurite outgrowth and cell growth and survival with respect to insulin. We cultured Lrrk2 deficient and wild-type mouse primary hippocampal neurons in the absence or presence of additional insulin and compared neurite outgrowth between both genotypes and different media conditions. In presence of additional insulin and concurrent absence of hFGF (−hFGF/+insulin) the Lrrk2 deficient hippocampal neurons developed significantly longer neurites than wild-type cells (p-value 0.0005, Suppl. Fig. 1d). This difference was abolished in media containing hFGF without insulin addition (+hFGF/−insulin) (Suppl. Fig. 1d). In the absence of both (hFGF and insulin) almost all neurons died 24 h after plating, independent of genotype, and the few survivor cells are stunted and apoptotic.

Next we analysed a possible effect of insulin on cell growth and survival of hematopoietic precursors using the CFC assay. We found an insulin-dependent “better” survival of BFU-Es/CFU-GEMMs from Lrrk2 deficient mice (p = 0.0287) compared to those of wild-type littermates in media containing additional insulin and without Epo (Suppl. Fig. 1e). In media without additional insulin no significant growth advantages were seen (Suppl. Fig. 1e, p = 0.164).

Taken together our data show impaired regulation of the insulin signalling pathway, especially Akt-dephosphorylation (=feedback control) in Lrrk2 deficient cells. This defect is not restricted to a single animal model or cell type.

### Lrrk2 deficiency affects insulin dependent GLUT4 translocation to the cell surface

**Figure 4:**
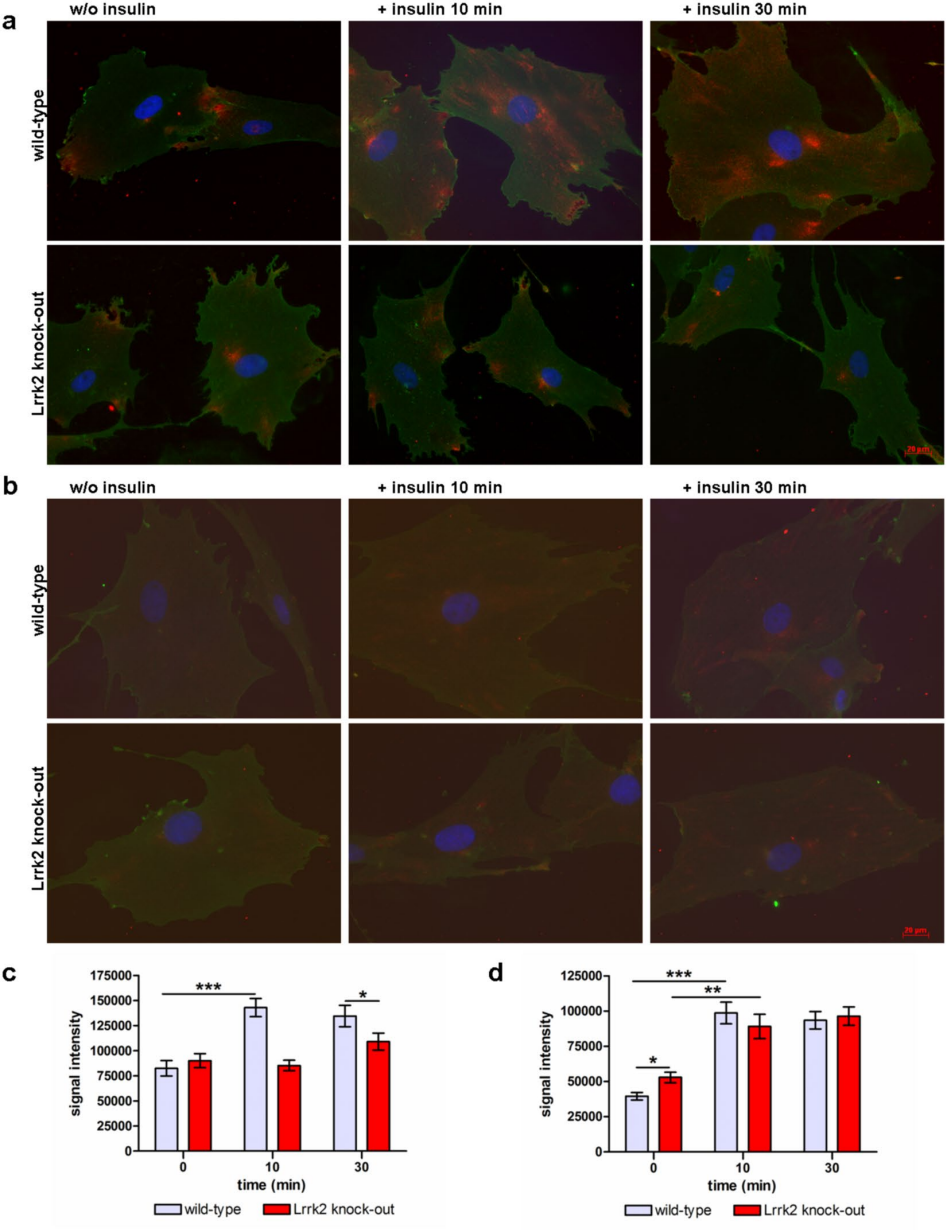
GLUT4 translocation in Lrrk2 deficient fibroblasts. (**a**) GLUT4 immunostaining on the cell surface of fibroblasts from 6 months old Lrrk2 deficient and wild-type rats without (w/o) insulin and 10 and 30 min after insulin addition and (**c**) the corresponding quantification of the GLUT4 signal intensity at different time-points (0, 10 and 30 min) after stimulation (mean and SEM). (**b**) GLUT4 immunostaining on plasma membrane of fibroblasts from 22 months old Lrrk2 deficient and wild-type rats without insulin and 10 and 30 min after stimulation and (**d**) the corresponding quantification of the GLUT4 signal intensity (mean and SEM). Red: GLUT4 immunostaining; green: wheat germ agglutinin (=plasma membrane) staining; blue: DAPI.

**Figure 5:**
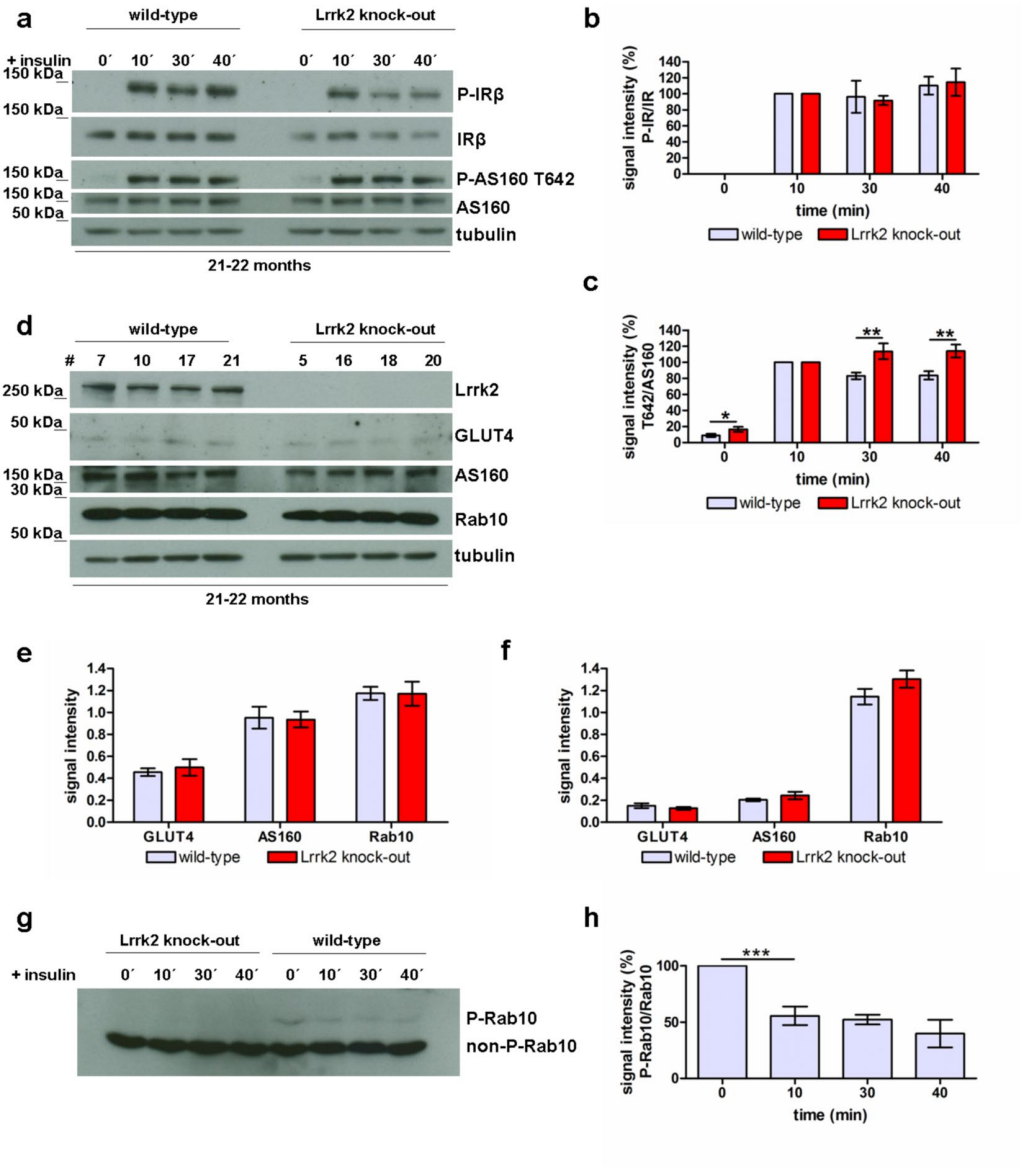
Insulin signalling in Lrrk2 deficient animals. (**a**) Insulin-triggered phosphorylation of IRβ and AS160 (Thr642) in fibroblasts from 22 months old Lrrk2 deficient rats at different time-points after stimulation and the corresponding quantification of P-IRβ (**b**, n = 7, normalized to IR-β) and P-AS160 Thr642 (**c**, n = 10, normalized to AS160) signal intensity (mean ± SEM). (**d**) Western blot analysis (fibroblasts from 22 months old rats as example) and quantification of total GLUT4, AS160 and Rab10 expression in fibroblasts from 6 months (**e**) and 22 months old (**f**) Lrrk2 deficient and wild-type rats (normalized to tubulin, mean ± SEM). # are numbers of animals/cell lines. The difference in GLUT4 and AS160 signal intensity between 6 months und 22 months old sample-groups results from differences in experimental procedure and does not reflect the absolute quantity of GLUT4 or rather AS160 in these age groups. (**g**) Investigation of Rab10 phosphorylation by Mn^2+^ Phos-tag SDS-PAGE in fibroblasts from 6 months old Lrrk2 deficient and wild-type rats at different time points (0-10-30-40 min) after insulin addition and (**h**) corresponding quantification of P-Rab10 signal intensity in wild-type cells at different time points after stimulation (normalized to Rab10, n = 7, mean and SEM).

Addition of insulin triggers glucose uptake in cells through the glucose transporter GLUT4. Under low extracellular insulin levels most of the GLUT4 is stored in intracellular vesicles. A rise in extracellular insulin induces rapid translocation of GLUT4 containing vesicles to the plasma membrane and their subsequent fusion with it (reviewed in)^55–57^. GLUT4 is primarily expressed in adipose tissues and striated muscle but also in fibroblasts^58^. Following our previous observations we investigated insulin-dependent GLUT4 translocation in Lrrk2 deficient fibroblasts by immunocytochemistry (ICC). In fibroblasts from 6 months old rats a distinct increase of GLUT4 immunostaining on the cell surface in the presence of insulin (after 10 and 30 min) is detected compared to non-stimulated cells (Fig. 4a (top) and c). Fibroblasts from 6 months old Lrrk2 deficient rats did not show any significant insulin-dependent changes in GLUT4 expression on the plasma membrane, the immunostaining-intensity of GLUT4 stays almost unchanged over time (0–30 min) with a slight up-regulation after 30 min (Fig. 4a (button) and c). Interestingly, we did not find this difference in insulin-dependent GLUT4 distribution in fibroblasts from 22 months old wild-type and Lrrk2 deficient rats: Fibroblasts from both genotypes show comparable intensity of GLUT4 immunostaining on the plasma membrane after insulin stimulation (Fig. 4b,d). Notably, GLUT4 immunostaining on the cell surface of fibroblasts from 22 months old Lrrk2 deficient rats at time-point 0 min (=without stimulation) is significantly increased in comparison to wild-type control cells (p-value 0.0056, Fig. 4d). To look at total GLUT4 expression we performed Western blotting of protein lysates from fibroblasts of 6 and 22 months old rats. Lrrk2 deficient and wild-type cells express comparable total amounts of GLUT4 (p-value 0.4654 for 6 months and 0.4295 for 22 months old animals, Fig. 5d–f).

### LRRK2 controls insulin signal transduction through phosphorylation of Rab10

Insulin signalling is initiated by insulin binding to the insulin/IGF1-receptor which in turn undergoes autophosphorylation and catalyses phosphorylation of downstream proteins (reviewed in)^59,60^. Based on our insulin related data for the activity of Akt we investigated expression and dynamics of insulin receptor (IR) phosphorylation using Western blot analysis. We do not find any alterations in activation of IRβ in Lrrk2 deficient mouse and rat cells (Fig. 5a,b, Suppl. Fig. 3e,f). Next we analysed the dynamics of the primary positive response which signals from activated IR downstream to Akt. As measured by the intensity of phospho-Akt Thr308 and Ser 473 signals at the beginning of stimulation (time points 0 and 10 min), the increase of Akt phosphorylation induced by insulin is comparable in Lrrk2 deficient and wild-type cells (Suppl. Fig. 3g). Downstream of Akt, phosphorylation of AS160 is a crucial step for regulation of insulin-dependent GLUT4 translocation^61–67^. Multiple Akt phosphorylation sites have been identified on AS160 *in vivo*. Subsequently, we analysed the phosphorylation of AS160 at Thr642 in Lrrk2 deficient fibroblasts by Western blotting. In comparison to wild-type we found a temporally prolonged and elevated phosphorylation at Thr642 in cells from 22 months old Lrrk2 deficient rats (p-value 0.0071 and 0.008 at 30 and 40 min after stimulation respectively, Fig. 5a,c). Furthermore, we found a significant (p = 0.0488) up-regulation in phosphorylation at Thr642 before stimulation (time-point 0 min) in fibroblasts from 22 months old Lrrk2 deficient rats (Fig. 5c), which is in agreement with our data for increased GLUT4 immunostaining at time point 0 (Fig. 4d). This up-regulation of “basal” AS160-phosphorylation in cells from 22 months old Lrrk2 deficient rats seems to be independent from Akt activity: we did not find any alterations in “basal” phosphorylation at Akt-Thr 308 and Akt-Ser437 in Lrrk2 deficient cells (=before stimulation, at time point 0, Fig. 3a–d). The total expression of AS160 is comparable in both genotypes as showed by Western blotting (Fig. 5d–f).

AS160 has a functional Rab GTPase-activating protein (GAP) domain. The GAP domain of AS160 displays strong activity toward Rabs 2 A, 8 A, 10 and 14^68^. Rab8A and especially Rab10 have been implicated in GLUT4 translocation^63,69^. Both Rab GTPases have been described as possible physiological substrates for LRRK2 *in vivo* and *in vitro*^39^. Using a Mn^2+^ -Phos-tag SDS-PAGE we analysed and compared insulin-dependent phosphorylation of Rab10 in fibroblasts from wild-type and Lrrk2-deficient rats. In concordance with published data^39^ we found a complete absence of phosphorylated Rab10 signal in Lrrk2 deficient cells (Fig. 5g), supporting the notion that Rab10 is a putative substrate for LRRK2. Furthermore, by comparison of Rab10 signal intensities and mobility shifts in protein-extracts from wild-type rat fibroblasts we found a significant difference in phosphorylation between un-stimulated and insulin-stimulated samples: phosphorylation of Rab10 decreases 10 min after insulin addition (p-value 0.0003, n = 7 independent experiments, Fig. 5g,h). As tested by Western blotting, the total expression of Rab10 is comparable in wild-type and Lrrk2 deficient cells (Fig. 5d–f, p-value are 0.562 and 0.271 for fibroblasts from 6 and 22 months old rats respectively).

### Insulin signalling in fibroblasts from Parkinson’s patient with G2019S mutated LRRK2

**Figure 6:**
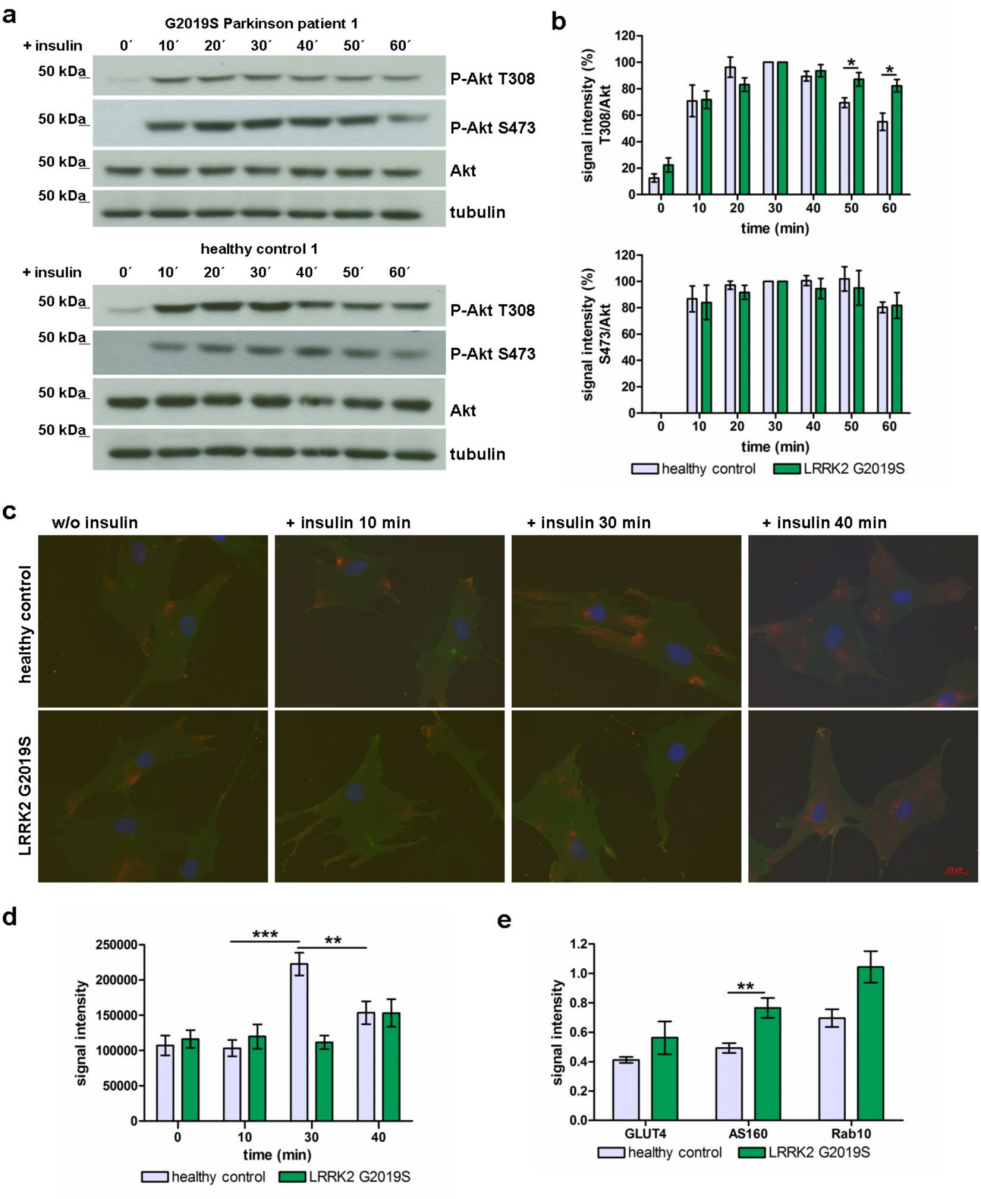
Insulin signalling in human fibroblasts from Parkinson’s patients with G2019S mutated LRRK2. (**a**) Example: Western blot analysis of protein extracts from fibroblasts of one PD patient with G2019S mutation (top) and one healthy control individual (button) and (**b**) the corresponding quantification of P-Akt Thr308 (top) and P-Akt Ser473 (button) signal intensity (normalized to Akt, n = 7 independent experiments, fibroblasts from 3 different healthy controls and 3 PD patients with G2019S mutation in LRRK2; mean ± SEM). (**c**) GLUT4 immunostaining on the plasma membrane of human fibroblasts derived from PD patients with G2019S mutation and healthy control individuals (red: GLUT4 immunostaining; green: wheat germ agglutinin (=plasma membrane) staining; blue: DAPI) and (**d**) the corresponding quantification of GLUT4 signal intensity (mean ± SEM). (**e**) Quantification of total GLUT4, AS160 and Rab10 protein expression in human fibroblasts (normalized to tubulin) analysed by Western blot (mean and SEM).

Finally, we aimed to translate our findings from Lrrk2 deficient animals to human cells. We used fibroblasts with a known pathogenic LRRK2 mutation and investigated insulin signal transduction. First, we looked at dynamic intracellular Akt phosphorylation and GLUT4 on the cell surface of human fibroblasts from PD patients with the G2019S mutation at different time points after stimulation with insulin. If compared to cells from age- and sex-matched healthy controls we found a significantly elevated phosphorylation of Akt at position Thr308 in G2019S mutated fibroblasts 50 and 60 min after addition of insulin (p = 0.0444 and 0.0105 respectively, Fig. 6a,b (top)). Phosphorylation on Ser473 is not affected at any time points tested (Fig. 6a,b (button)). By immunofluorescence microscopy we found a slight non-significant increase of GLUT4 staining on the cell surface of G2019S mutated patient cells 40 min after stimulation (Fig. 6c,d). In contrast, fibroblasts from healthy control individuals show a strong, significant (p-value 0.000001) elevation of GLUT4 immunostaining 30 min after insulin addition followed by a distinct decline of staining within next ten minutes (p-value 0.0015, time point 40 min, Fig. 6c,d). The total GLUT4 expression is comparable in fibroblast from LRRK2 G2019S PD patients and healthy controls as shown by Western blot analysis (p-value 0.2983, Fig. 6e and Suppl. Fig. 3h). Investigation of AS160 and Rab10 protein expression in human fibroblasts showed a significant up-regulation (p-value 0.0035) for AS160 and slight but non-significant (p = 0.055) elevation for Rab10 (Fig. 6e and Suppl. Fig. 3h).

## Discussion

Within this study we investigated the possible role of the Parkinson’s disease-linked LRRK2 in intracellular signalling and showed that LRRK2 plays a crucial role in insulin-dependent signal transduction. Insulin-induced translocation of glucose transporter type 4 containing vesicles to the cell surface fails under Lrrk2 deficiency as can be seen by the strong reduction of GLUT4 immunostaining of plasma membranes in cells from 6-months old Lrrk2 deficient animals. Furthermore, using the Phos-tag SDS-PAGE we find that the LRRK2-dependent phosphorylation of Rab10 GTPase seems necessary und crucial for sufficient insulin-triggered GLUT4 translocation. Further investigations in cells from 22-months old Lrrk2 deficient animals showed considerable basal and insulin dependent changes in phosphorylation of the key signalling protein Akt, phosphorylation and expression of Akt substrate AS160, and the cellular distribution of GLUT4. These molecular compensatory arrangements appear to restore GLUT4 translocation deficits in Lrrk2 deficient cells at higher age as can be seen using ICC. In addition we find that the Lrrk2-dependent changes in phosphorylation of Akt seems to be restricted to insulin signal transduction: the dynamics of Akt phosphorylation triggered by hFGF, IL-3, IL-6, M-CSF and C5a in Lrrk2 deficient cells is not affected. Furthermore, our data indicate a functional insulin/IGF1-receptor and unhindered downstream insulin signalling up to AS160 in Lrrk2 deficient cells.

GLUT4 is the insulin-regulated glucose transporter found primarily in adipose tissues and striated muscle (skeletal and cardiac). Recent reports demonstrated the expression of the *GLUT4* (*SLC2A4*) gene in fibroblasts^58^ and parts of the central nervous system such as the hippocampus. Moreover, impairment in insulin-stimulated trafficking of GLUT4 in the hippocampus results in decreased metabolic activities and plasticity of hippocampal neurons, which leads to depressive like behaviour and cognitive dysfunction^70–72^.

Insulin-triggered transport of glucose into cell is mediated by the redistribution of GLUT4 from intracellular GLUT4 storage vesicles (GSVs) to the plasma membrane^73^. The redistribution of GLUT4 involves insulin signal transduction and vesicle trafficking, each of which is comprised of multiple steps. It is well established that activation of insulin/IGF1-receptor by insulin and subsequent activation of PI3K through insulin receptor substrate is essential for insulin-dependent GLUT4 translocation (reviewed in)^60^. Downstream from PI3K, the phosphorylation of PKB/Akt and its substrate AS160 is required and sufficient to trigger GLUT4 translocation^60,62,66,74,75^. In addition to the PI3K/Akt pathway, other signalling intermediators have been implicated in the regulation of GLUT4 translocation^76–78^. The GLUT4 trafficking pathway itself includes budding of GSVs from intracellular compartments, transport of GSVs along cytoskeleton (microtubules and actin filaments), docking, priming and fusion at the plasma membrane (summarized in)^56^.

The investigation of the insulin signal transduction in Lrrk2 deficient cells revealed significant dys-regulation in PI3K/Akt pathway and expression of AS160. AS160 phosphorylation by Akt in response to insulin regulates its interaction with 14-3-3^79^. Insulin-stimulated phosphorylation and 14-3-3 binding inhibit the AS160 GAP activity, which leads to activation of Rabs and GLUT4 translocation. 14-3-3 proteins have been described as LRRK2 interactors^43–49^. Furthermore, binding of 14-3-3 proteins to LRRK2 is known to be impaired by PD-relevant LRRK2 mutations R1441C/G/H, Y1699C and G2019S^46,48^. Using a Reactome pathway analysis tool Porras *et al*.^80^ showed a potential role of LRRK2 in GLUT4 translocation via interaction with 14-3-3 proteins. A possible inhibition of AS160 - 14-3-3 interaction by Lrrk2 deficiency or/and mutations in LRRK2 and subsequent increased AS160 GAP activity are supposable mechanism for affected insulin-triggered GLUT4 translocation in cells from Lrrk2 deficient animals and Parkinson’s patients with G2019S mutation.

The GAP domain of AS160 displays strong activity toward Rabs 8A, 10 and other Rab GTPases^68^. Rab8A and especially Rab10 are required for insulin-stimulated translocation of GLUT4 vesicles^63,69,81^ and have been described as direct physiological substrates for LRRK2 *in vivo* and *in vitro*^39^. LRRK2 directly phosphorylates these on evolutionary conserved residues Thr72 and Thr73 respectively in the switch II domain^39^. By Phos-tag SDS-PAGE we confirm the Rab10 phosphorylation by LRRK2 *ex vivo*, as the phospho-specific Rab10 signal lacks completely in Lrrk2 deficient cells. Furthermore, we found a significant insulin-triggered effect on Rab10 phosphorylation in wild-type fibroblasts: the phosphorylation-signal is decreased by ca. 50% 10 min after insulin addition. Therefore we conclude that (i) the presence of LRRK2 is essential for Rab10 phosphorylation; (ii) the phosphorylation of Rab10 by LRRK2 is necessary for sufficient GLUT4 translocation to the cell surface and (iii) the activation of the insulin pathway triggers an unknown insulin-dependent phosphatase X responsible for subsequent dephosphorylation of Rab10. The continuous phosphorylation of Rab10 by LRRK2 and the insulin regulated switch to the non-phosphorylated form seems to be a crucial mechanism for efficient GLUT4 vesicle translocation.

**Figure 7:**
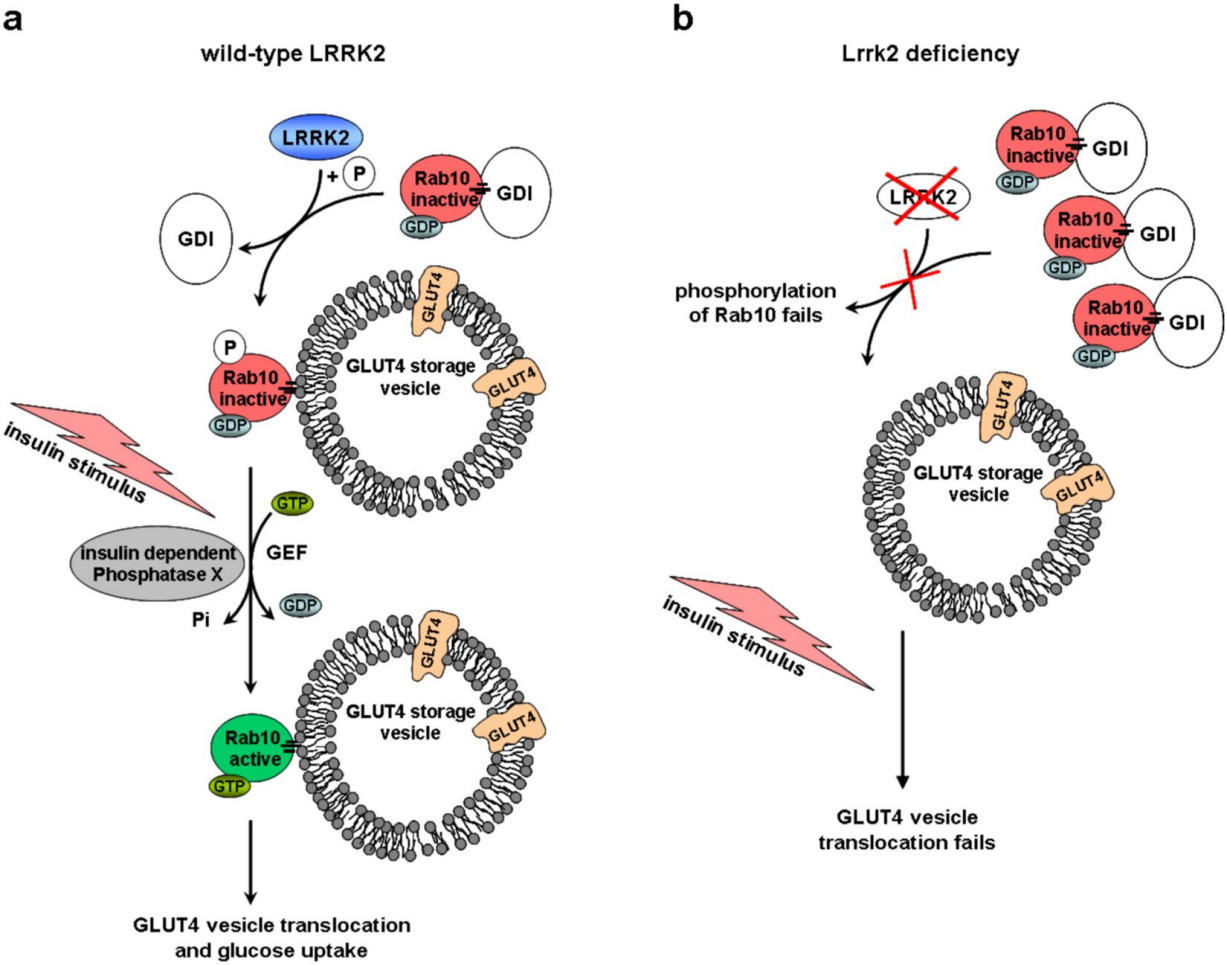
The role of LRRK2 for insulin signal transduction. (**a**) In wild-type, GDP-bound Rab10 is tightly bound by guanine dissociation inhibitor (GDI) in the cytosol. LRRK2 promotes the insertion of Rab10 in GLUT4 storage vesicles by phosphorylation. Insulin addition activates an insulin-dependent Phosphatase X with following dephosphorylation of Rab10 (accompanied by GDP-GTP exchange), necessary for efficient GLUT4 translocation. (**b**) In LRRK2 deficient situation, the phosphorylation of Rab10 by LRRK2 and the following insertion in GLUT4 storage vesicles fail. As consequence, GDP-bound Rab10 – GDI complexes accumulate in the cytosol. By insulin addition activated PI3K/Akt/AS160 signalling cascade did not reach the GLUT4 storage vesicles and the GLUT4 translocation fails.

More than 60 Rab GTPases are involved in almost every step of vesicle-mediated transport (summarized in)^82^. Rabs are reversibly associated with membranes. Guanine dissociation inhibitors (GDIs) extract inactive Rabs from membranes and bind them in the cytoplasm (summarized in)^83^. Interference of the Rab-GDI interaction through affected phosphorylation results in an altered subcellular distribution of Rab GTPases: non-phosphorylatable Rab mutants accumulate in the cytosol, phosphomimetric mutant associate with the membrane fraction^39^. Therefore, a disrupted membrane-cytosol distribution of Rab10 in Lrrk2 deficient cells is quite imaginable: abolished phosphorylation of Rab10 results in its accumulation in the cytoplasm; Rab10-targeting to the GLUT4 storage vesicle (GSV) membrane fails (Fig. 7). Using the standard fluorescence immunocytochemistry (ICC) we tried to investigate insulin triggered sub-cellular re-distribution of Rab10 and its co-localisation with GSVs in rat fibroblasts. Unfortunately, insulin-regulated GSVs cannot be easily seen, because GLUT4 resides in small, insulin-responsive and non-insulin-responsive GSVs as well as larger structures that are derived from endosomes and trans-Golgi network. Distinguishing between all these compartments using fluorescence microscopy is extremely difficult, if not impossible (technical and biological challenges are summarised in)^57^. In our approach we did not find any clearly visible differences in Rab10 distribution between wild-type and Lrrk2 deficient cells or between stimulated and non-stimulated cells (data not shown).

Lrrk2 deficiency is accompanied by significantly elevated phosphorylation of Akt and AS160 as well as increased expression of GLUT4 on the cell surface in “aged” cells. These molecular changes, with the exception of slightly elevated Akt phosphorylation, are not characteristic of “young” cells and therefore are a secondary compensatory response, possibly induced by sugar uptake deficit. Our data suggest that this intracellular “compensatory strategy” starts in young animals and reach gradually its maximum in aged individuals. On the one hand the dys-regulated protein expression and phosphorylation “rescues” the GLUT4 translocation to the cell surface in Lrrk2 deficient cells. On the other hand undesirable secondary effects by prolonged Akt phosphorylation are inevitable and affect cell homeostasis accompanied by long-term consequences. Affected development and survival of Lrrk2 deficient hematopoietic stem cells (Fig. 2d) and hippocampal neurons (Fig. 2a,c) are some examples for disrupted control of intracellular signalling in Lrrk2 deficient cells. Altogether our data provides evidence that LRRK2 deficiency in presence of insulin can outflank the otherwise strict regulation of cellular processes by growth factors, cytokines and other factors.

Rab10 studied here is just one of many Rab GTPases described to interact with LRRK2^39^. Rab3, Rab5, Rab7, Rab8, Rab12, Rab32 are published as further substrates and interaction partners for LRRK2 (summarized in^17,37,39,40^), very probably even more Rabs interact. As already indicated, Rab GTPases are important for almost every step of vesicle-mediated transport of any kind, therefore the involvement of LRRK2 in a number of different intracellular processes based on vesicular transport is not surprising (synaptic vesicle recycling/endocytosis^21–24^; mitochondrial- and autophagy/lysosomal functions^17,27,29–31,34^ and others). The molecular mechanism regulating most (if not all) of these processes are possibly based on phosphorylation of the target Rab GTPases by LRRK2 and followed by extracellularly or intracellularly triggered de-phosphorylation, or similar processes, and need to be investigated in additional studies.

The characterization of protein expression and phosphorylation in human fibroblasts derived from PD patients with the pathogenic G2019S mutation indicate significant changes for Akt and AS160, similar to those in Lrrk2 deficient cells. Timmons *et al*.^54^ showed that defective Akt is implicated as a putative signalling pathway linked to loss of dopaminergic neurons in PD. The presence of the hyperactive G2019S mutated LRRK2 in the cell shifts the balance towards phosphorylated Rab GTPases and results in accumulation of Rabs in membranes^39^. Follow-up investigations are needed to clarify the exact molecular mechanism behind these intracellular changes.

The discussion about the interactions between PD and diabetes mellitus began in the 1960’s and there is still controversy. It has been reported that 50 to 80% of patients with PD have abnormal glucose tolerance (summarized in)^84^. Several clinical studies show a correlation between Parkinson’s disease and diabetes: Patients with diabetes have a high risk (up to 60% higher than healthy controls, dependent on the population under study) of Parkinson’s disease onset^85–87^. Mebel *et al*.^88^ show a significant reduction of dopamine-dependent signal transduction in the brain by insulin via increased reuptake of dopamine by dopamine transporter (DAT). Dopamine depletion in the striatum can cause a decrease of insulin signalling in basal ganglia; several therapeutic targets for PD (AMP-activated protein kinase, glucagons-like peptide-1 etc., summarized in)^89^ reinforce the association of PD with diabetes^89^.

Overall the insulin signalling pathway might play an important role in disease pathogenesis of LRRK2 associated PD and might have potential therapeutic implications in the future. Additional clinical studies (e.g. impact of “low carbohydrates” diet on PD-progression) are needed to clarify therapeutic power of insulin signal suppression in PD.

## Methods

### Ethical approval and informed consent

Primary human cells were isolated from skin-biopsies. Cell isolation was approved by the ethical commission of the University of Tuebingen (Application 287/2004V). Informed and written consent from all patients and healthy individuals was obtained. Experiments were conducted in compliance with the rules for investigation on human subjects, as defined in the Declaration of Helsinki.

Animal experiments were approved by the Regional Commission of Tuebingen and conducted with strict accordance with the European Convention for the Protection of Vertabrates used for Scientific Purposes.

### Animal models

The Lrrk2 knock-out mouse was generated as described^51^. The Lrrk2 knock-down mouse was generated by recombination of the neo-U6shLrrk2 donor construct via phiC31 integrase into the modified *Rosa* locus of the IDG3.2 mouse ES cell line (Fig. 1b) and the derivation of germline chimaeric mice^52^. The shRNA was addressed to a 25 nt target sequence within the Lrrk2 mRNA (sense: GGACAGCTTTCCTTATTTGACTTAA, antisense: TTAAGTCAAATAAGGAAAGCTGTCC, separated by a 8 bp loop: GAAGCTTG).

The Lrrk2 knock-out rat was generated in our lab via microinjection of a pair of TAL-nucleases into pronuclei. TAL-nucleases were directed against target sequences within exon 2 of *Lrrk2* (TALEN1: TGATAGTCCTGGACT; TALEN2: TACCTGCTGCACACT) and constructed as described^90^. The rat line used here contains a 7 bp deletion in exon 2 of the *Lrrk2* gene (Fig. 1a) resulting in an open read frame shift and translational stop. All mouse and rat models were kept as heterozygous lines (two independent lines (lines 1 and 2) for each animal model) by breeding to C57BL/6 mice (Charles River) or CD (SD) rats (Charles River). For experiments, the heterozygous animals were bred to generate homozygous Lrrk2 deficient mice and rats. Wild-type littermates were used as wild-type control in all experiments.

### Primary neuronal culture

Pups from heterozygous breeding were prepared at postnatal day 0 (P0). Brains were removed and transferred to a culture dish containing sterile HBSS (Gibco/Invitrogen). Brain hippocampi were dissected under a microscope and transferred to a 1.5 ml tube containing HBSS. For enzymatic digestion, the HBSS was replaced by 0.05% (w/v) Trypsin/EDTA (Gibco, 25300-054) and the tissue/Trypsin suspension was kept at 37 °C for 14 min (water bath). The tissue fragments were washed three times with 1 ml Neurobasal-A/Glutamax I media (Gibco/Invitrogen) and mechanically digested by trituration using a yellow pipet tip. The appropriate amount of the cell suspension was mixed with culture media (Neurobasal-A with Glutamax I (Gibco/Invitrogen), 1x B27 Supplement (Gibco/Invitrogen) and growth factors). The cells were seeded on poly-DL-ornithine hydrobromide (Sigma, P-8638) coated 96-well imaging plates (BD Falcon, 353219) or 6-well plates (Nunc). The media was replaced on the next day and then every two days. Cultures were maintained at 37 °C, 5% CO_2_. The following growth factors were used for this study: rhFGF (5 ng/ml), rhBDNF (5 ng/ml), rmEpo (5 ng/ml) and rrCNTF (5 ng/ml), all from R&D Systems. To study physiological consequences of Lrrk2 deficiency in respect to insulin the culture media was supplemented with insulin-free B27 supplement (Gibco/Invitrigen).

### Colony forming cell assay

The methylcellulose based colony forming cell (CFC) assay was performed according to the manufacturers’ protocol (http://www.rndsystems.com/literature_MouseMethylcellulose.aspx). For the assay a suspension of hematopoietic cells was prepared by mechanical trituration of spleen from 3–4 months old mouse. The cells were washed twice with IMDM (12440, Gibco/Invitrogen) and cultivated for 10 days at 37 °C and 5% CO_2_ in medium HSC006 (R&D Systems) without any cytokines (negative control) and medium HSC008 (R&D Systems) containing mSCF, mIL-3, mIL-6, human insulin and human transferrin without Erythropoietin (Epo) or with different concentrations of Epo (rmEpo, #959-ME, R&D Systems).

### Antibodies

Primary antibodies used: Akt #9272 (1:1000), p-Akt (Ser473) # 4058 (1:1000), p-Akt (Thr308) #2965, Rab8A #6975 (1:1000), Rab10 #8127 (1:1000), AS160 #2670 (1:1000), p-AS160 (Thr642) #4288 (1:1000), IR beta #3025 (1:1000), p-IGF-IR beta (Tyr1131)/IR beta (Tyr1146) #3021 (1:1000) (all from Cell Signalling); LRRK2 (3514-1 (MJFF2) Epitomics); tubulin (ab6160, abcam); SLC2A4 (ARP43785_P050, avivasysbio, 1:100); GLUT4 (MAB1262, R&D Systems, 1:1000); Alexa Fluor 488 Mouse anti-β-Tubulin, Class III Clone TUJ1 #560338 (BD Pharmingen).

Secondary antibodies used: anti-rabbit IgG, HRP-linked #7074 (1:5000, Cell signalling); Goat anti-rat IgG, HRP-linked (1:20 000, ab6845, abcam); Rabbit anti-sheep IgG, HRP-linked (1:10 000, 31480, ThermoScientific); Goat anti-mouse IgG, HRP-linked (1:10 000, 172–1011, Bio-Rad); Cy3-conjugated Goat anti-rabbit IgG (1:350, 111-165-046, Jackson ImmunoResearch); Cy2-conjugated Goat anti-mouse IgG (1:350, 115-225-164, Jackson ImmunoResearch).

### Immunocytochemistry

For the neurite outgrowth quantification, cells were stained with Alexa Fluor 488 Mouse anti-β-Tubulin, class III antibody (BD Pharmingen, 560338) according to the manufacturer´s protocol for bioimaging. In brief, cells were fixed with 3.7% (w/v) paraformaldehyde (Electron Microscopy Sciences, 15710), washed three times with PBS (Biochrom, L1825), permeabilized with 0.1% Triton X-100 and block with 3% (v/v) NGS in PBS for 30 min. Cells were stained with 50 µl/well anti-β-Tubulin, class III antibody (1:50 in PBS) plus DAPI (0.5 ng/ml) for 1 h at RT, and washed three times with PBS. Finally, cells were analysed using BD Pathway 855. The neurite outgrowth quantification occurred on AttoVision 1.5 software (BD Bioscience).

For GLUT4 distribution analysis fibroblasts were seeded on 10 mm glass coverslips over night. On next day the media was replaced to RPMI1640 (Biochrom, F1275) with 1xGlutaMAX I (Gibco, 35050) for 2 h. For insulin stimulation the media was replaced again to PBS (Biochrom, L1825). Human recombinant insulin (biomol, 87402) was diluted in HBSS with calcium and magnesium (Gibco, 14025) containing 0.1% (w/v) BSA (final dilution 1 µg/ml) and add to the cells for the indicated time. Cells were fixed with 4% (w/v) paraformaldehyde (Electron Microscopy Sciences, 15710), washed with PBS (Biochrom, L1825) and stained with Wheat Germ Agglutinin, Alexa Fluor™ 488 Conjugate (W11261, Thermo Fisher Scientific) in PBS (final concentration 5 µg/ml) for 10 min at room temperature. Next the cells were washed, blocked with 15% (v/v) NGS in PBS for 1 h and stained with anti-SLC2A4 antibody (1:100 in 5% (v/v) NGS in PBS) over night at 4 °C. After washing with PBS coverslips were mounted using Vectashield mounting medium (Vector Laboratories, H-1500). Finally, cells were analysed using a fluorescence microscope (Zeiss Axio Imager Z1). The fluorescence intensity was quantified using ImageJ.

### Western blot analysis

For tissue protein extracts, animals were killed by CO_2_ and the tissue homogenized in 3x volumes (w/v) of ice-cold lysis-puffer (PBS containing 0.1% Triton X-100, protease inhibitor (Roche, 11836170001) and phosphatase inhibitor (Roche, 04906837001) cocktails). Tissue homogenates were incubated for 15 min on ice and centrifuged at 13.000 g, 4 °C for 15 min. The supernatant was aliquotted and stored at −80 °C.

For cell protein extracts, cells from a 6-well were lysed in 75 µl ice-cold lysis-puffer, incubated on ice for 5 min and lysates centrifuged at 13.000 g, 4 °C for 15 min. Protein concentration was measured by BCA protein assay (Pierce). For Western blot analysis, 50–100 µg of the tissue protein lysates or 25–50 µg of the cell lysates were mixed with 2x Laemmli sample buffer (Sigma, S3401-10VL), separated in 8% or 8–12% gel (peqlab PerfectBlue Twin) and transferred to Immobilon-P PVDF membrane (0.45 µm, IPVH00010, Millipore). Membranes were blocked in 5% (w/v) milk (A0830, AppliChem) in TBS-T for 1 h at RT, incubated with primary antibody overnight at 4 °C and for 1 h with secondary HRP-conjugated antibody. For detection, membranes were incubated with ECL detection solution (ECL plus or ECL prime western blot detection reagents, Amersham/GE Healthcare) and signal was detected using X-ray film (Amersham Hyperfilm ECL). For the quantification of the signal intensity, the chemiluminescence signal was detected by a CCD camera (FluorChem 8900 imaging system, AlphaInnotech) and the intensity was measured using AlphaEaseFC software (AlphaInnotech) or ImageJ.

### Mn^2+^ Phos-tag SDS-PAGE

Cell lysates prepared as described in “Stimulation with insulin and growth factors” were mixed with 2x Laemmli sample buffer (Sigma, S3401-10VL) containing 0.5 nM MnCl_2_, heated at 95 °C for 5 min and loaded on a gel. Gels for Phos-tag SDS-PAGE consisted of a stacking gel (4.5% acrylamide (Mix 29:1, AppliChem, A0951), 125 mM Tris/HCl pH 6.8, 0.1% (w/v) SDS, 0.1% (v/v) TEMED (Roth) and 0.05% (w/v) ammonium persulfate (APS)) and a separating gel (10% acrylamide (Mix 29:1, AppliChem, A0951), 376 mM Tris/HCl pH 8.8, 0.1% (w/v) SDS, 50 µM Phos-tag acrylamide (Aviva Systems Biology), 0.1 mM MnCl_2_, 0.1% (v/v) TEMED and 0.05% (w/v) APS). Samples were electrophoresed using running buffer (25 mM Tris base, 192 mM glycine, 0.1% (w/v) SDS) at 18 mA for the stacking part and 36 mA for the separating part (2 gels, BioRad Mini Trans-Blot Cell System). Gels were washed three times for 10 min in the transfer buffer (48.5 mM Tris base, 39 mM glycine, 20% (v/v) methanol, 0.04% (w/v) SDS) containing 10 mM EDTA with gentle agitation. Next, gels were soaked in the transfer buffer without EDTA for 10 min and proteins transferred to the PVDF membrane (0.2 µm, BioRad, 162–0177). After transfer membranes were blocked with 5% (w/v) milk (A0830, AppliChem) in TBS-T for 1 h at RT, incubated with primary antibody diluted in 5% (w/v) BSA in TBS-T overnight at 4 °C and for 1 h with secondary HRP-conjugated antibody. For detection, membranes were incubated with ECL detection solution (ECL prime western blot detection reagents, Amersham/GE Healthcare) and signal was detected using X-ray film (Amersham Hyperfilm ECL). For the quantification, the signal intensity was measured using AlphaEaseFC software (AlphaInnotech) or ImageJ.

### Bone marrow derived monocyte isolation and culturing

The monocyte precursors were isolated from mice or rat bone marrow of the hind limbs by centrifugation of the bone marrow cell mix through a percoll (GE Healthcare, 17-0891-02) density gradient (3 ml 45%, 5 ml 62% and 3 ml 81% (v/v) percoll in HBSS). The cell fraction between 45 and 62% layers containing monocyte precursors was cultured in IMDM media (21056-023, Gibco by life technologies) containing 20% (v/v) FCS, 1% (v/v) Pen/Strep and 100 ng/ml M-CSF (R&D Systems) at 37 °C, 5% CO_2_.

### Fibroblasts isolation and culturing

Mouse and rat fibroblasts were isolated from ear pieces. Ear pieces were cut in small parts, put in 2–3 wells of a 6-well plate (Nunc) containing 50 µl fibroblast culture media (RPMI 1640 (Millipore, F1215), 20% (v/v) FCS (Gibco by life technologies, 10270), 1% (v/v) Pen/Strep (Millipore, A2213), 10 mM HEPES (Gibco by life technologies, 15630-056), 1 mM Sodium pyruvate (Millipore, L0473), 1x NEAA (Millipore, K0293), 1x Glutamax-I (Gibco by life technologies, 35050-038)), covered with 2 coverslips and flooded with 3 ml fibroblasts culture media. The media was changed every 3–4 days. Growing fibroblasts were split using Accumax solution (Sigma, A7089) after the cells become confluent. For all experiments fibroblasts in the passage 3 to 7 were used.

All human fibroblasts from Parkinson patients and healthy control individuals are from the Blood- and Tissue-library Hertie Institute for Clinical Brain Research Tuebingen (Ethics-Application 287/2004V). Human fibroblasts are cultured in RPMI media (Millipore, F1215) containing 10% (v/v) FCS (Gibco by life technologies, 10270), 1 mM Sodium pyruvate (Millipore, L0473) and 1x Glutamax-I (Gibco by life technologies, 35050-038).

### Stimulation with insulin and growth factors

For Western blot analysis cells were plated on 24-well culture plates (Nunc, monocytes: 70 000/well; fibroblasts: 50 000/well) and cultured over night. Before stimulation, media was replaced with growth factor free and low serum (2% (v/v) FCS) media for 2 h. Subsequently, the media was replaced to PBS and insulin (or growth factors) diluted in HBSS with Ca^2+^ and Mg^2+^ containing 0.25% (w/v) BSA were added. Final concentrations for insulin (human, recombinant, biomol, # 08563): 1 µg/ml; IL-3 (R&D Systems, 403-ML): 10 ng/ml; IL-6 (R&D Systems, 406-ML): 10 ng/ml; M-CSF (R&D Systems 416-ML-050 and 216-MC-025): 100 ng/ml; C5a (recombinant mouse C5a, R&D Systems, # 2150-C5): 10 ng/ml. At different time-points the PBS/HBSS stimulation mix was removed and the cells lysed in 30 µl ice-could lysis puffer (PBS containing 1% (v/v) Triton X100, Protease inhibitor cocktail (Roche, # 04 693 124 001) and Phosphatase inhibitor cocktail (Roche, # 04 906 845 001)) for 5 min on ice. Protein lysates were centrifuged at 13.000 g, 4 °C for 15 min and stored at −80 °C or directly separated in gel (s. Western blot analysis and Mn^2+^ Phos-tag SDS-PAGE).

### Statistical analysis

The data were analysed by two-tailed Wilcoxon-Mann-Whitney U-test and two-tailed t-test.

## Supplementary information

Supplementary Informations
