# Supplementary material for: The Parkinson’s disease-linked Leucine-rich repeat kinase 2 (LRRK2) is required for insulin-stimulated translocation of GLUT4
Source: Sci Rep. 2019 Mar 14;9:4515. doi: 10.1038/s41598-019-40808-y (PMC6418296; doi:10.1038/s41598-019-40808-y)
Supplement: Supplementary file 1 — Supplementary Informations [file 41598_2019_40808_MOESM1_ESM.pdf]

The Parkinson´s disease-linked Leucine-rich repeat kinase 2 (LRRK2) is required for insulin-stimulated translocation of GLUT4

Natalja Funk[1]*, Marita Munz[1], Thomas Ott[2], Kathrin Brockmann[1], Andrea Wenninger-Weinzierl[1], Ralf Kühn[3,4], Daniela Vogt-Weisenhorn[4], Florian Giesert[4], Wolfgang Wurst[4,5], Thomas Gasser[1], and Saskia Biskup[1]*.

[1] Hertie Institute for Clinical Brain Research and German Center for Neurodegenerative Diseases, University Clinic Tuebingen, Tuebingen, Germany

[2] IZKF Facility for Transgenic Animals, Institute of Medical Genetics and Applied Genomics, University of Tuebingen, Tuebingen, Germany

[3] Max-Delbrueck-Center for Moleculare Medizin and Berlin Institute of Health, Berlin, Germany

[4] Helmholtz Zentrum Muenchen, Technical University Muenchen-Weihenstephan, Institute of Developmental Genetics, Neuherberg, Germany

[5]German Center for Neurodegenerative Diseases, Munich, Munich Cluster for Systems Neurology (SyNergy), Munich, Germany

* Corresponding authors: natalja.funk@medizin.uni-tuebingen.de; saskia.biskup@medizin.uni-tuebingen.de

Saskia Biskup, Hertie Institute for Clinical Brain Research and German Center for Neurodegenerative Diseases, Otfried-Mueller Str. 27, 72076 Tuebingen, Germany.

Supplementary Figures.

Supplementary Figure 1.

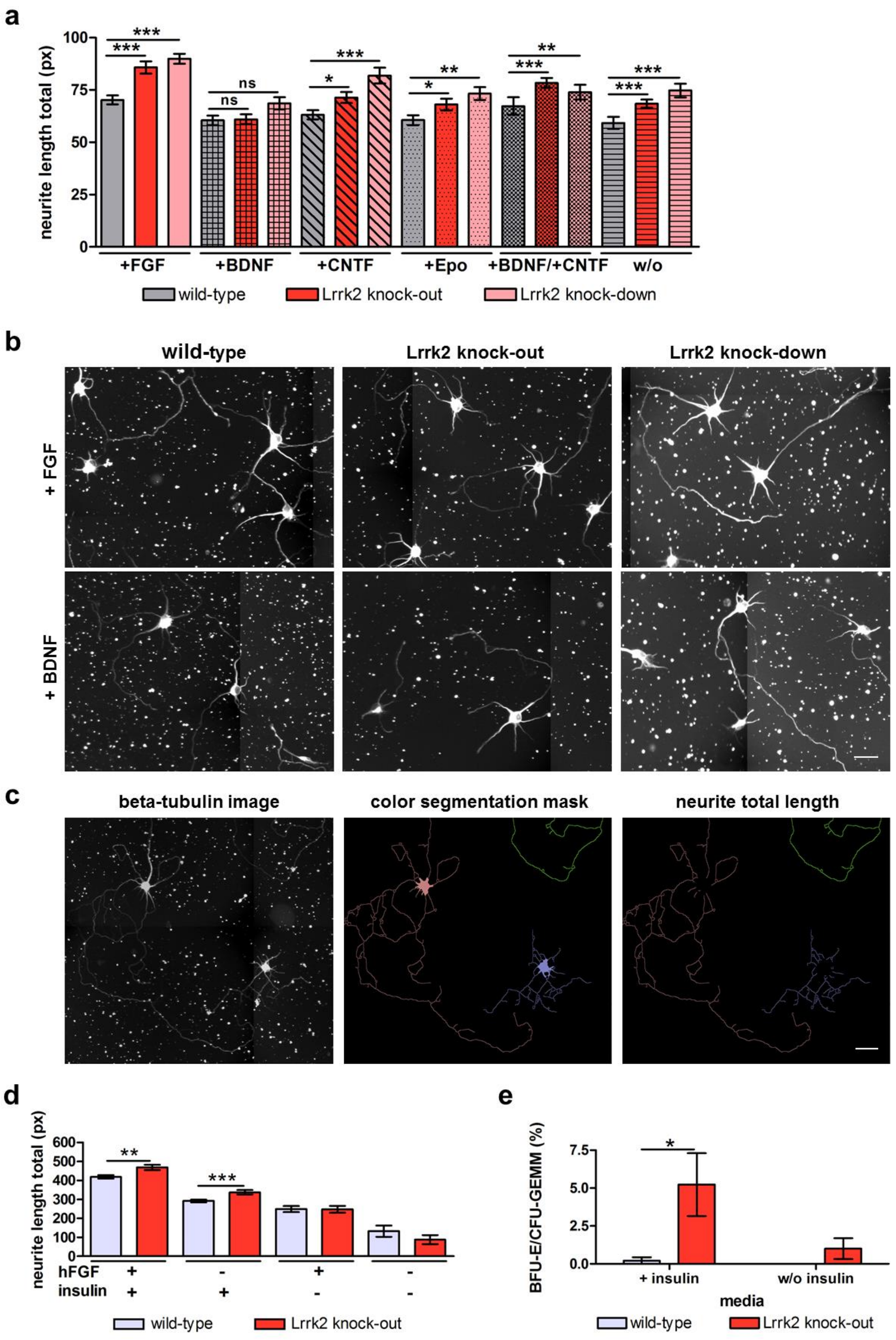

Supplementary Figure 2.

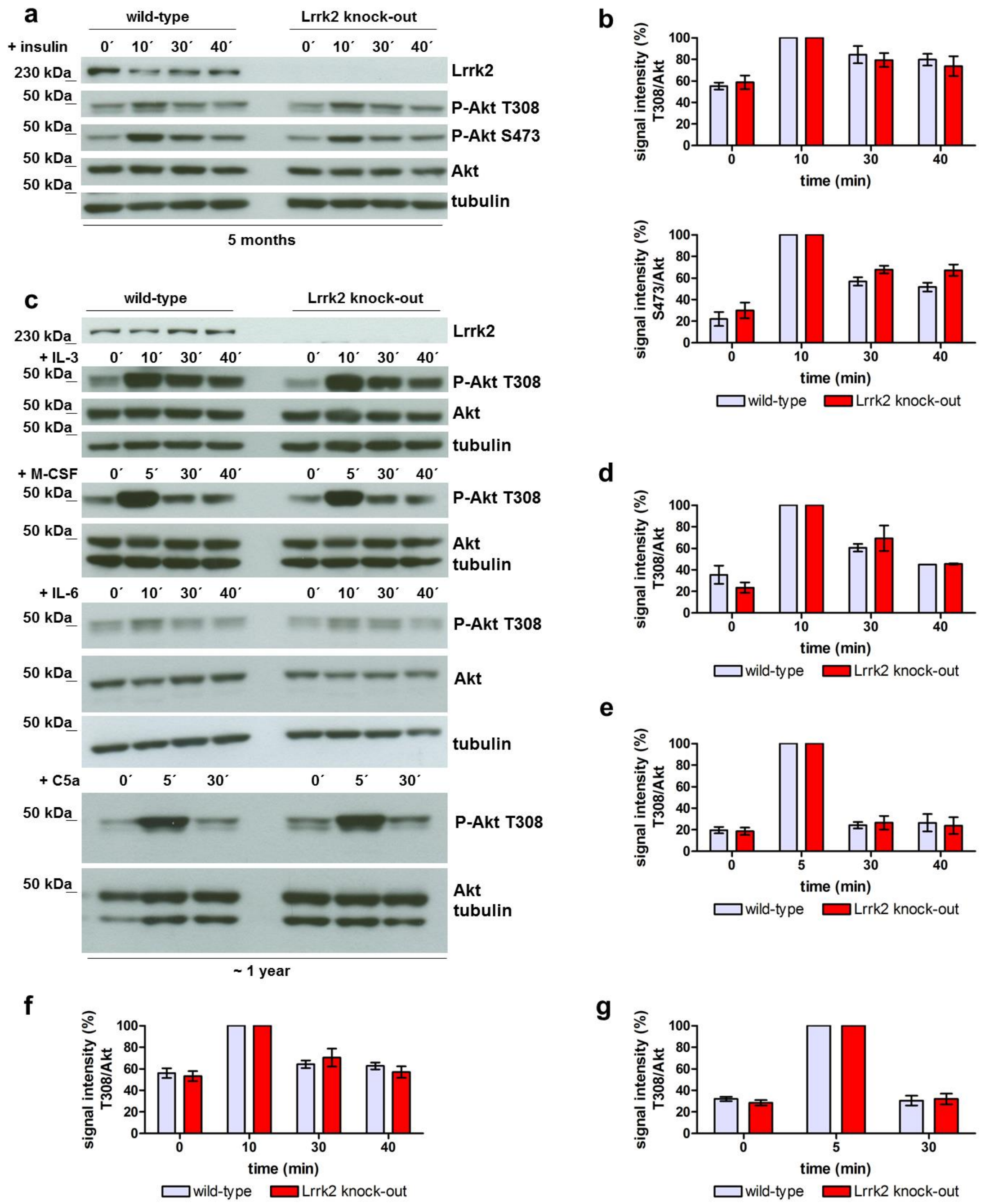

Supplementary Figure 3.

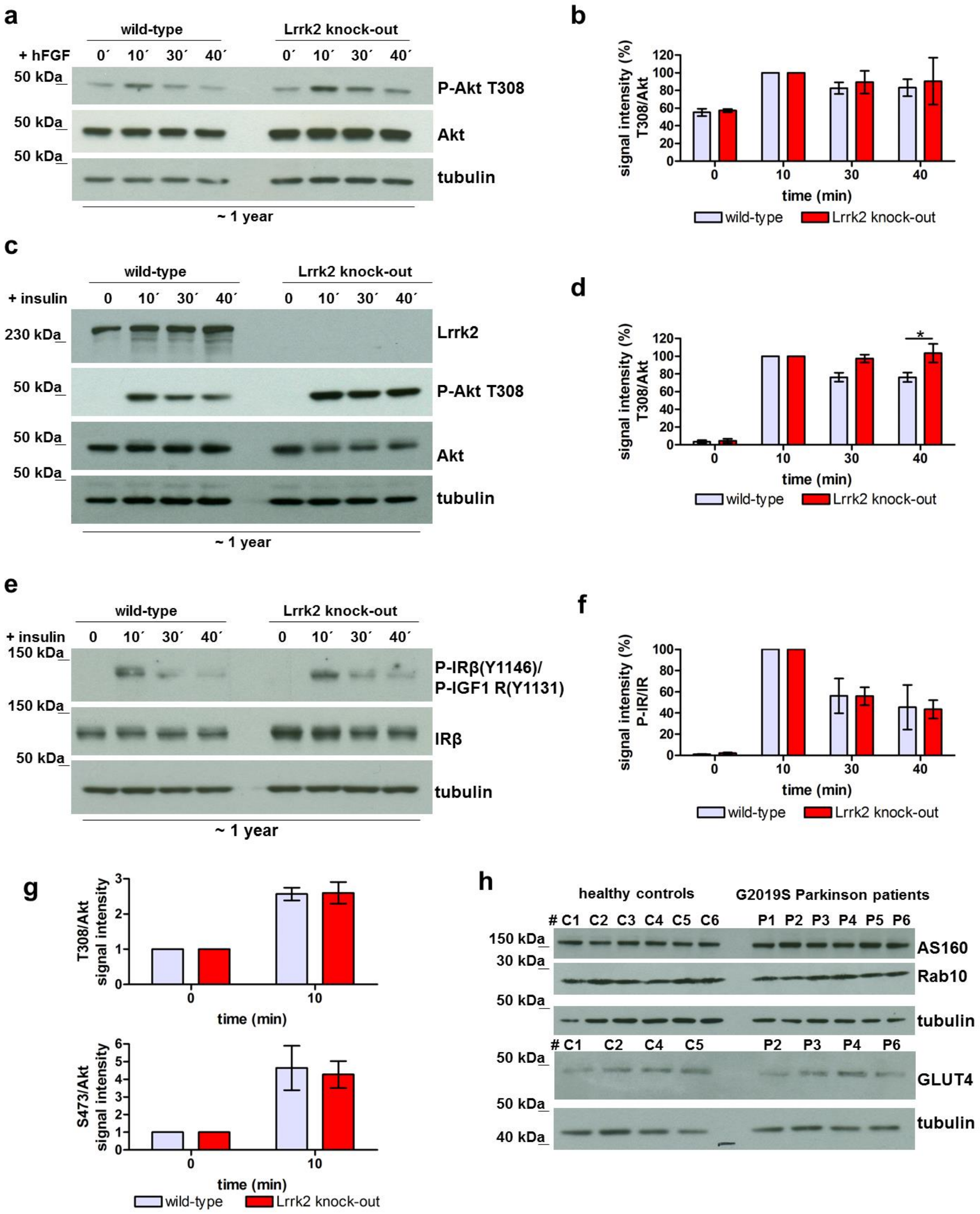

Supplementary Figure 1. (a) Total neurite length of mouse wild-type, Lrrk2 knock-out and Lrrk2 knock-down hippocampal neurons in presence of different growth factors and without any growth factors (w/o) at DIV1. (b) Representative pictures of mouse wild-type, Lrrk2 knock-out and Lrrk2 knock-down hippocampal neuron cultures at DIV3 stained with anti-β-Tubulin class III antibody TUJ1 used for investigation of neurite outgrowth by high throughput microscopy and (c) a corresponding segmentation mask used for analysis. Scale bars: 20 px. (d) Neurite outgrowth of Lrrk2 deficient and wild-type mouse primary hippocampal neurons in the absence or presence of additional insulin (DIV 3; mean and SEM). (e) CFC-Assay: The effect of insulin on cell growth and survival of hematopoietic precursors (BFU-Es/CFU-GEMMs) from Lrrk2 deficient and wild-type mice. N = 9 animals/genotype, two-tailed t-test, mean and SEM.

Supplementary Figure 2. (a) Western blot analysis of Akt-phosphorylation at Thr308 and Ser 473 in monocytes from 5 months old Lrrk2 deficient and wild-type mice at different time points after addition of insulin and corresponding quantification of P-Akt Thr308 (b (top), n = 6) as well as P-Akt-Ser473 (b (button), n = 7) signal intensity (normalized to Akt, mean ± SEM). (c) Western blot analysis of phospho-Akt T308 in protein extracts from monocytes of 1 year old Lrrk2 deficient and wild-type mice at different time points after stimulation with IL-3, M-CSF, IL-6 and C5a and corresponding quantification of P-Akt T308 signal intensity for IL-3 (d), M-CSF (e), IL6 (f) and C5a (g), normalized to Akt, mean ± SEM.

Supplementary Figure 3. (a) Western blot analysis of P-Akt Thr308 in fibroblasts from 1 year old Lrrk2 deficient and wild-type mice at different time-points (0 – 40 min) after stimulation with hFGF and (b) the corresponding quantification of P-Akt Thr308 signal intensity (normalized to Akt, mean ± SEM). (c) Akt-phosphorylation in fibroblasts from 1 year old Lrrk2 deficient and wild-type mice at different time-points

after stimulation with insulin and (d) the corresponding quantification of P-Akt Thr308 signal intensity (n = 7, normalized to Akt, mean ± SEM). (e) Insulin triggered phosphorylation of IRβ/IGF1 R in monocytes from 1 year old Lrrk2 deficient and wild-type mice at different time points after insulin addition and (f) the corresponding quantification of P-IRβ/P-IGF1 R signal intensity (n = 6, normalized to IRβ, mean ± SEM). Wild-type and Lrrk2 deficient mice monocytes showed comparable dynamic of IRβ phosphorylation. (g) Increase of P-Akt Thr308 (top, n = 8) and P-Akt Ser473 (button, n = 7) signal intensity in monocytes from 1 year old wild-type and Lrrk2 knock-out mice 10 min after insulin addition (mean ± SEM, normalized to Akt). (h) Western blot analysis of total GLUT4, AS160 and Rab10 expression in human fibroblasts. # are numbers of healthy controls (C1-C6) and Parkinson´s patients (P1-P6).
